# The label-free detection and distinction of CYP2C9-expressing and non-expressing cells by surface-enhanced Raman scattering substrates based on bimetallic AuNPs–AgNWs

**DOI:** 10.1039/c9ra02046b

**Published:** 2019-04-30

**Authors:** Xiaowei Cao, Shuai Chen, Zhenyu Wang, Yong Liu, Xiaowei Luan, Sicong Hou, Wei Li, Hongcan Shi

**Affiliations:** Institute of Translational Medicine, Medical College, Yangzhou University Yangzhou 225001 PR China weili@yzu.edu.cn shihongcan@yzu.edu.cn; Jiangsu Key Laboratory of Integrated Traditional Chinese and Western Medicine for Prevention and Treatment of Senile Diseases, Yangzhou University Yangzhou 225001 PR China; Jiangsu Co-innovation Center for Prevention and Control of Important Animal Infectious Diseases and Zoonoses, Yangzhou University Yangzhou 225009 China; Jiangsu Key Laboratory of Experimental & Translational Non-coding RNA Research Yangzhou 225001 PR China; The Key Laboratory of Syndrome Differentiation and Treatment of Gastric Cancer of the State Administration of Traditional Chinese Medicine Yangzhou 225001 PR China; School of Life Science and Medicine, Dalian University of Technology Panjin 124221 China

## Abstract

Cytochrome P450 2C9 (CYP2C9) is capable of catalyzing the biotransformation of endogenous compounds in cells, indicating that this enzyme could change the intracellular environment and is related to the pathogenesis of diseases. Currently, it is still a challenge to study the differences in cellular components between CYP2C9-expressing and non-expressing cells. In this study, employing a Au nanoparticles–Ag nanowires (AuNPs–AgNWs) decorated silicon wafer as a novel non-destructive and label-free tool, we applied surface-enhanced Raman scattering (SERS) spectroscopy to detect and distinguish the cellular composition of CYP2C9-expressing cells (293T-Mig-2C9) and non-expressing cells (293T-Mig-R1). AgNWs with high surface roughness were formed by modification of AuNPs onto their surface by electrostatic interactions, which enabled them to exhibit greatly enhanced SERS ability. Then, they were employed to fabricate SERS substrates *via* an electrostatically assisted 3-aminopropyltriethoxysilane (APTES)-functionalized surface-assembly method. The SERS substrates exhibited high sensitivity with a detection limit of 1 × 10^−9^ M for 4-mercaptobenzoic acid (4-MBA). Meanwhile, the SERS substrates exhibited good uniformity and reproducibility. The cytotoxicity assay demonstrated that the SERS substrates displayed good biocompatibility with 293T cells. Before SERS measurements, CYP2C9 constantly expressed cells (293T-Mig-2C9 cells) and control cells (293T-Mig-R1 cells) were constructed. The expression of CYP2C9 and the catalytic activity in the cells were checked. Using the AuNPs–AgNWs substrates as a high-performance *in vitro* sensing platform allowed us to obtain fingerprint spectra of 293T-Mig-R1 and 293T-Mig-2C9 cells. The difference spectra between the two cell lines were studied to interpret the spectral differences and gain insight into the biochemical variations. Finally, principal component analysis (PCA) score plots of the SERS spectra were also used to better view the differences between the two cell lines. SERS detection based on the AuNPs–AgNWs substrates provides a sensitive, non-destructive and label-free method for differentiation between 293T-Mig-R1 and 293T-Mig-2C9 cells.

## Abstract 2

A AuNPs–AgNWs decorated silicon wafer was used as a non-destructive and label-free tool in SERS spectroscopy to detect and distinguish the cellular composition of CYP2C9-expressing cells (293T-Mig-2C9) and non-expressing cells (293T-Mig-R1).

## Graphical abstract

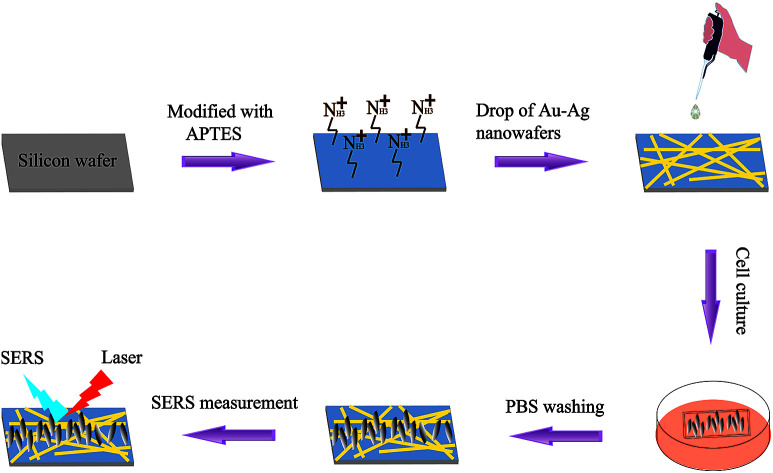

## 1. Introduction

Cytochrome P450 2C9 (CYP2C9) belongs to the cytochrome P450 superfamily, which are mainly responsible for mono-oxygenate reactions. Besides being primarily expressed in human liver and small intestine, CYP2C9 proteins were also detected in the colon, kidney, lung, heart, skin, blood vessels and other tissues.^1,2^ The well-characterized function of CYP2C9 is that it is responsible for drug biotransformation.^3,4^ Moreover, CYP2C9 also modulates endogenous compounds, including catalyzing the metabolism of arachidonic acid and steroids,^5,6^ as well as the generation of reactive oxygen species.^7^ Based on the findings mentioned above, we could speculate that this enzyme may be involved in the renin–angiotensin–aldosterone system or tumorigenesis.^8^ Therefore, understanding how CYP2C9 proteins alter the intracellular microenvironment is of critical importance for the investigation of substance metabolism in cells and pathogenesis of related diseases. However, detection of and discrimination between CYP2C9-expressing and non-expressing cells in a noninvasive and label-free manner are still a challenge.

Surface enhanced Raman scattering (SERS) has been widely used as an analytical technique and is based on amplified Raman scattering with the help of plasmonic structures. When molecules are placed onto a metallic nanostructure's roughened surface, the SERS signal could be greatly enhanced by the existence of “hot spots” in the interstitial region.^9,10^ SERS could supply molecular information with high sensitivity for specific vibration modes that is also known as a “fingerprint”.^11^ Besides, SERS has lots of advantages, for example, being non-destructive, showing weak water scattering and involving minimal sample preparation compared with other analytical techniques.^12^ Therefore, it is a powerful tool for studying living cells in systems containing water. Up to this date, several published reviews have covered the application of SERS in cell detection. For example, Wang *et al.*^13^ described EGF-functionalized SERS probes for direct detection of circulating tumor cells in peripheral blood. Huefner *et al.*^14^ employed nuclear-targeted gold nanoparticles as intracellular SERS probes for the distinction of differentiated cell types in a human neuroblastoma cell line. Hodges *et al.* constructed SERS probes with gold-conjugated antibodies combined with silver enhancement to detect keratan sulphate chains on the cell surface of corneal endothelial cells. Compared with the conventional light microscopy techniques, this technique has been proved to be far more sensitive.^15^ In our previous reports, we developed intracellular SERS probes based on the trans-activator of transcription (TAT)-functionalized Au nanostars for label-free detection of the epithelial–mesenchymal transition process in alveolar epithelial type II cells.^16^ Besides, SERS has been used to identify apoptotic and necrotic cell death.^17^

SERS enhancement effects mainly result from “hot spots”, which originate from the rough surface of nanoparticles and the gaps between metallic nanoparticles. To gain reliable SERS measurement, the substrate materials are vital. Au nanoparticles–Ag nanowires (AuNPs–AgNWs) are of considerable current interest because of their highly tunable optical properties.^18^ Silver and gold nanostructures possess distinctive absorption peaks in the visible and near-infrared regions, and also their optical properties could be tuned by shape and dimension. Moreover, the coupling of plasmons between Au and Ag nanostructures separated by a nanoscale gap is considered to be important for amplification of SERS.^19,20^ The local electrical fields of the “hot spots” vary drastically because of the coupling between the Localized Surface Plasmon Resonances (LSPRs) of the nanoparticles and nanowires. Wei *et al.* demonstrated that the SERS effect could be greatly enhanced when the incident light was polarized across the AuNPs–AgNWs junction for the coupling between the plasmons in the nanowire and adjacent nanoparticle.^21^ However, the distance between them where nearly no SERS effect is generated has been estimated to be ∼200 nm.^22^ To achieve a higher enhancement effect, the AuNPs–AgNWs can be assembled to generate a higher number of “hot spots”. Xu *et al.*^23^ utilized a vacuum thermal evaporation and solid-state ionics method to synthesize AuNPs–AgNWs with high surface roughness. The Raman enhancement factor (EF) of the AuNPs–AgNWs substrates was up to 10^17^, which could be ascribed to the densely arranged “hot spots” located at the nanoparticle–nanowire junction. Therefore, to fabricate SERS nanostructures, such as bimetallic nanowires, novel designs should be sought.

**Scheme 1:**
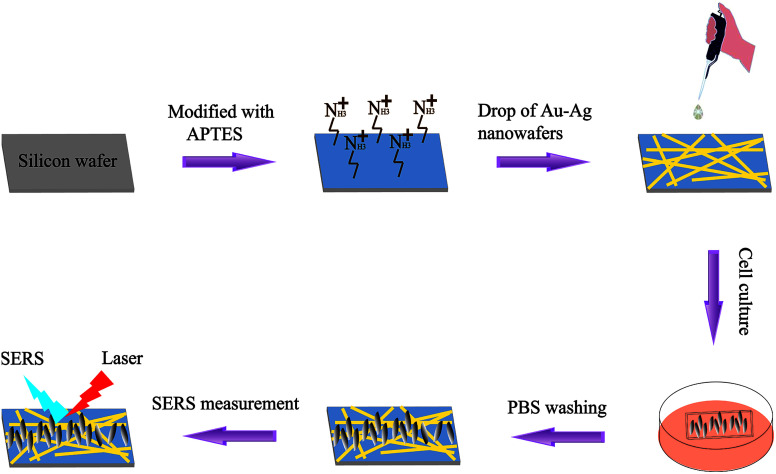
A schematic illustration of the assembly of AuNPs–AgNWs substrates and SERS used for discrimination between 293T-Mig-R1 and 293T-Mig-2C9 cells.

In this work, SERS was exploited to identify CYP2C9-expressing cells (293T-Mig-2C9) from non-expressing cells (293T-Mig-R1) by using silicon wafer-based substrates decorated with AuNPs–AgNWs (Scheme 1). Firstly, a facile electrostatic interaction method was used to prepare the AuNPs and Ag nanowires coupled as AuNPs–AgNWs in high yields. Secondly, the AuNPs–AgNWs were assembled onto the surface of (3-aminopropyl)triethoxysilane-(APTES-) functionalized silicon wafer. The SERS uniformity, sensitivity and reproducibility of the AuNPs–AgNWs substrates were all evaluated. Before SERS measurements, we constructed CYP2C9 constantly expressed HEK293T cells (293T-Mig-2C9 cells) and empty plasmid control cells (293T-Mig-R1 cells) by using MigR1 retroviral vector. Using AuNPs–AgNWs substrates as a high-performance *in vitro* sensing platform, the SERS spectra of the two cell lines were collected. The differences between 293T-Mig-2C9 cells and 293T-Mig-R1 cells were well compared and distinguished through difference spectral analysis and principal component analysis (PCA). AuNPs–AgNWs SERS substrates featuring rapid, label-free, and high sensitivity could serve as a promising tool for cell research, and the method shows potential for applications in high-throughput analysis, drug screening, and cell physiological process monitoring.

## 2. Materials and methods

### 2.1 Materials

Chloroauric acid tetrahydrate (HAuCl_4_·4H_2_O), sodium borohydride (NaBH_4_), ethylene glycol (EG), silver nitrate (AgNO_3_) and poly (vinyl pyrrolidone) (PVP K-30) were purchased from Yangzhou Younuo Chemicals Co., Ltd. (China). 4-MBA and APTES were purchased from Yangzhou Noah Chemical Co., Ltd (China). Ultrapure water used throughout the experiment was from Milli-Q (Millipore, America, resistivity > 18 M).

### 2.2 Synthesis of Ag nanowires

AgNWs were synthesized according to a modified Li's method.^24^ Briefly, 10 mL of EG was added into a 250 mL three-neck round-bottom flask at 160 °C for 1 h. 4 mL of AgNO_3_ (0.3 mM) EG solution was injected into the flask at the rate of 0.3 mL min^−1^ and 5 mL of PVP (0.2 mM) EG solution was injected into it at the same rate. The reaction was continued until the color of the solution turned light gray. Finally, the samples were washed with acetone twice and ethanol three times. PVP was used as both a soft template and surface stabilizer for the synthesis of the AgNWs, as it makes the surface of the AgNWs negatively charged.^25^

### 2.3 Synthesis of AuNPs–AgNWs

AuNPs were prepared by adding 20 mL of a NaBH_4_ (0.15 mM) solution to 50 μL of a HAuCl_4_ (24.3 mM) solution under vigorous stirring. After 40 min, the AuNP solution was stored at 4 °C. Then, 50 μL of the above solution of positive charged AuNPs was put into 1 mL of the negatively charged AgNW solution in a glass vial at room temperature under vigorous stirring. The solution was stirred for 4 h, and Au nanoparticles assembled on the surface of the AgNWs as much as possible. Finally, the as-prepared samples were washed with ethanol three times to get rid of the AuNPs that were not decorated onto the AgNWs. In this way, AuNPs–AgNWs could be produced.

### 2.4 Assembly of AuNPs–AgNWs substrates

The fabrication of the AuNPs–AgNWs substrates is displayed in Scheme 1. Briefly, silicon wafers were washed with aqua regia and then rinsed with ultrapure water three times. The wafers were further cleaned with ethanol three times, followed by drying for 2 h at 80 °C in an air oven. Then, the silicon wafers were immersed in a 1% (v/v) APTES ethanol solution for 12 h, which was followed by rinsing with anhydrous ethanol and air drying. Subsequently, the as-prepared solution containing AuNPs–AgNWs was directly dropped onto the APTES-functionalized silicon wafers. After the successful adsorption of AuNPs–AgNWs onto the surface of the APTES-functionalized silicon wafers, the wafers were washed with ultrapure water and dried. Thus, the SERS substrates assembled with AuNPs–AgNWs nanostructures were obtained.

### 2.5 Cell seeding and growth

HEK293T cells were obtained from the medical college of Yangzhou University. CYP2C9 constantly expressed HEK293T cells (293T-Mig-2C9 cells) and the empty plasmid control cells (293T-Mig-R1 cells) were constructed with MigR1 retroviral vector as previously described.^26^ The cells were cultured in DMEM medium, supplemented with 10% fetal bovine serum (Hangzhou Sijiqing Biological Engineering Materials Co., Ltd., Hangzhou, China), 100 U mL^−1^ penicillin and 0.1 mg mL^−1^ streptomycin (Beyotime Biotechnology, Shanghai, China). The prepared AuNPs–AgNWs substrates were immersed in 75% ethanol for 4 h and then were exposed to a high dose of UV light for 12 h to ensure adequate sterilization. 293T-Mig-2C9 cells or 293T-Mig-R1 cells and AuNPs–AgNWs substrates were dispensed in 6-well plates to guarantee *in situ* growth of cells on the surface of the substrate, respectively. After 24 h, the medium was removed. Subsequently, the cells were washed with PBS three times and finally selected for SERS measurements.

### 2.6 Detection of CYP2C9 expression and activity

The fluorescence of enhanced green fluorescent protein (eGFP) from Mig-Rl plasmid was detected with an EVOS FL Cell Imaging Systems instrument (ThermoFisher Scientific, WA, USA) to check the over-expression of the target gene. CYP2C9 protein expression was detected with western blotting assay. 293T-Mig-R1 and 293T-Mig-2C9 cells were lysed with RIPA buffer (Beyotime Biotechnology, Shanghai, China) and the protein concentration was detected with a BCA kit (Beyotime Biotechnology, Shanghai, China). 5 μg protein extracts were separated by SDS-PAGE and transferred to a nitrocellulose membrane. After blocking with 5% skimmed milk, the membrane was incubated with anti-CYP2C9 antibody (Bio-Rad Laboratories, Inc., CA, USA) and anti-β-actin antibody (ZSGB-BIO, Beijing, China). The membrane was washed and incubated with horseradish peroxidase conjugated anti-rabbit IgG or anti-mouse IgG antibodies (ZSGB-BIO, Beijing, China). The proteins were visualized by using an enhanced chemiluminescence detection system (Beyotime Biotechnology, Shanghai, China) and the results were documented with a Tanon 5500 Chemiluminescent Imaging System (Tanon, Shanghai, China).

CYP2C9 catalytic activity was detected with an ACQUITY UPLC System (Waters, MA, USA) coupled to a QTRAP 6500 triple quadrupole mass spectrometer (Applied Biosystems, CA, USA). Diclofenac (100 μM) was incubated with 293T-Mig-R1 or 293T-Mig-2C9 cells (2 × 10^6^) for 30 minutes. The reaction was terminated with an equal volume of ice-cold methanol containing warfarin sodium (0.5 μM) as an internal standard and then spun at 20 000 × *g*. The supernatant was separated with a 1.7 μm ACQUITY UPLC BEH C18 column (Waters, MA, USA). The mobile phase was 65% acetonitrile. Ionization was performed with an electrospray ionization (ESI) source in the negative mode. The multiple reaction monitoring (MRM) transitions for the metabolite (4′-OH diclofenac) and internal standard were 310.00 → 266.00 and 307.10 → 250.10, respectively.

### 2.7 CCK-8 viability assays

A CCK-8 kit was utilized to investigate the cytotoxicity of the AuNPs–AgNWs substrates on these two cell lines. Before the test began, the substrates were disinfected with a 75% (v/v) ethanol aqueous solution for 1 h and placed in a culture dish under aseptic conditions. Then, 1.0 × 10^4^ cells of each cell line were seeded onto the surface of each substrate in a culture dish to allow attachment. After incubating for 24 h, the medium was removed. 4 mL of fresh medium and 0.4 mL of CCK-8 solution were added and cultured for 2 h at 37 °C. The absorbance of the suspension was read at 450 nm. These two cell lines cultured in the culture dish without the substrates were used as a control. The cell viability was calculated as follows:
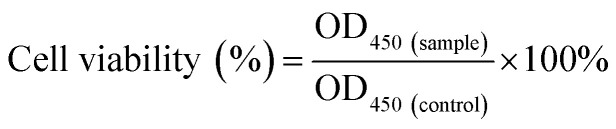


### 2.8 SERS measurement and data analysis

Before measurement, the wavelength position and instrumental response were calibrated using monocrystalline silicon at 520 cm^−1^. For the SERS measurement of AuNPs–AgNWs, 4-MBA was used as the analyte. SERS spectra were all recorded in the range of 600–1800 cm^−1^ using a Renishaw Invia microscope Raman spectrometer with a 785 nm He–Ne laser. The laser power focused on the samples was 5 mW and the exposure time was 10 s. In the detection of cells, SERS spectra of 293T-Mig-R1 and 293T-Mig-2C9 cells were measured by using a He–Ne laser with 1 mW power. The laser spot size was ∼1 μm from a 50× lens. The test cell samples were kept moist with PBS during data acquisition. SERS spectra of individual cells were obtained through the scan-excitation mode. In order to obtain spatial-averaged Raman spectral information of single cells, the laser spot was scanned across nearly the whole cell during Raman acquisition. Then, the measured SERS spectra of 20 cells from each cell line were used to obtain their mean SERS spectrum.

The spectral processing procedure of individual cells including 5-point smoothing, normalization and vertical movement was carried out using the software Micro Origin 8.0. The intensity of the cellular SERS spectrum was normalized to get a relative intensity from 0 to 1. Then, the comparison between the spectra of 293T-Mig-R1 and 293T-Mig-2C9 cells was made through the subtraction of different mean spectra and the shifts of the different peaks in the subtracted spectra were assigned to the molecular structures and biochemical components based on previous studies and the literature. Finally, Matlab version 8.1 software was used for PCA. PCA was performed on normalized spectra, and PC1 was determined by retaining the maximum variance (maximum information). Each subsequent principal component describes a maximum of variance that is not modeled by the former components, so most of the variance is contained in the first few principal components (PCs). All redundant information is summarized, thus simplifying the graphical interpretation of the data.

### 2.9 Characterization

The morphology of the as-prepared nanostructures was studied with a S-4800 II field-emission scanning electron microscope (SEM) and a Tecnai 12 transmission electron microscope (TEM) (Philips). The high-resolution transmission electron microscope (HRTEM) image and selected area electron diffraction (SAED) image were captured by using a Tecnai G2 F30 S-Twin TEM (FEI). The chemical composition of the AuNPs–AgNWs was also analyzed by using a S-4800 II field-emission scanning electron microscope. The optical density (OD) at 450 nm of CCK-8 was measured by using a Cary UV-5000 spectrometer.

## 3. Results and discussion

### 3.1 Characterization of Ag nanowires and AuNPs–AgNWs

**Fig. 1:**
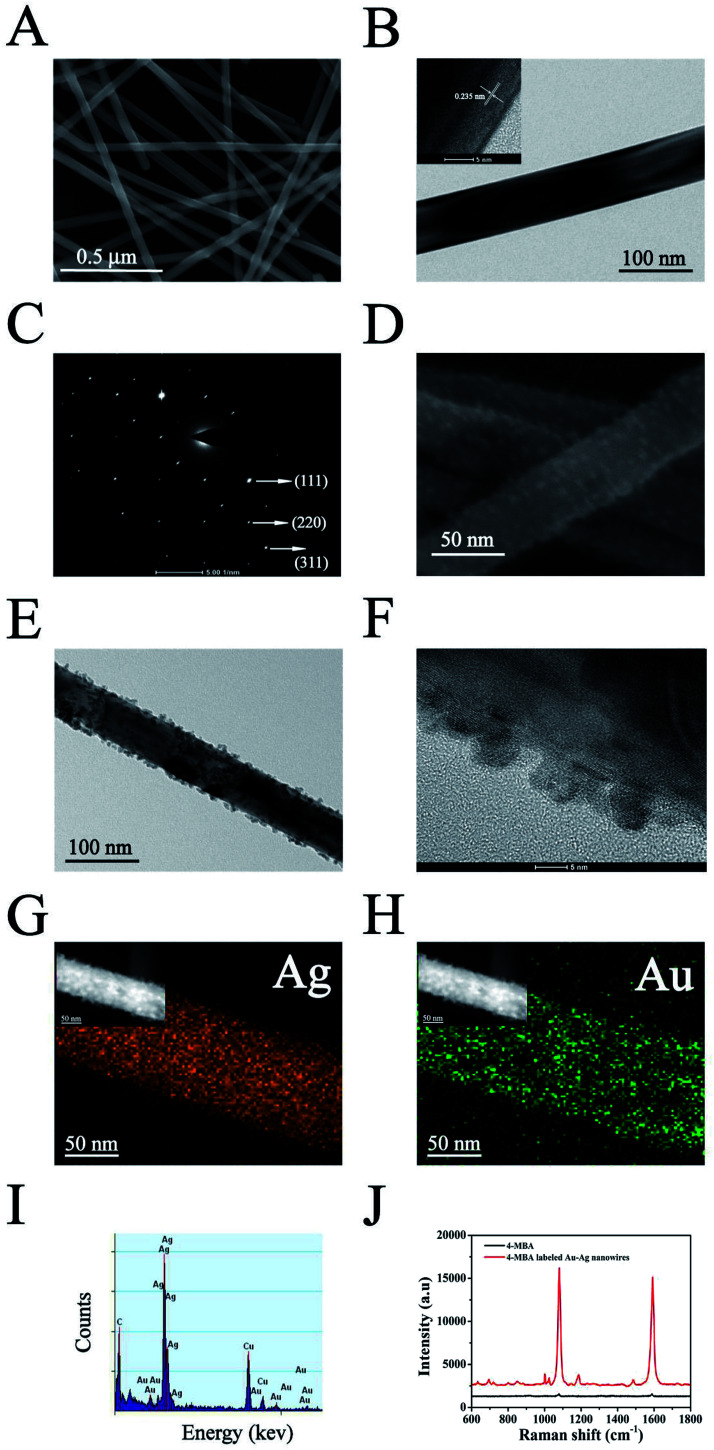
(A) SEM and (B) TEM images of AgNWs. The inset shows the HRTEM image of the AgNWs. (C) The SAED pattern of the AgNWs. (D) SEM, (E) TEM and HRTEM (F) images of the AuNPs–AgNWs. The related EDS elemental mapping of the AuNPs–AgNWs (G and H). (I) The EDS spectrum of the AuNPs–AgNWs. (J) Raman spectra of 4-MBA labeled AuNPs–AgNWs and 4-MBA solution.

The morphology and size of the bimetallic structure of the AgNWs and AuNPs–AgNWs were characterized by using a SEM and TEM. Fig. 1A reveals that the as-prepared AgNWs had a relatively high aspect ratio and the diameter was about 60 nm. Few Ag nanoparticles appeared, indicating that the yields of silver nanowires were high. The TEM image (Fig. 1B) shows that the surface of the AgNWs is smooth. To observe the crystalline structure of the AgNWs, a HRTEM image was captured. As shown in the inset of Fig. 1B, the interplanar distance was measured to be 0.235 nm, which matched the reflection from the Ag {111} crystal planes.^27^ The SAED pattern (Fig. 1C) of the AgNWs exhibited bright spots corresponding to the {111}, {220}, and {311} crystal planes, indicating the polycrystallinity of the AgNWs. AuNPs–AgNWs were prepared by assembling the positively charged AuNPs onto the surface of the negatively charged AgNWs by electrostatic interaction. Fig. 1D presents the morphologies of the Au–Ag hybrid nanowires. Compared with the smooth surface of the AgNWs, it was obvious that the surface of the assembled AgNWs became uneven due to the assembly of AuNPs. Fig. 1E shows the typical TEM images of AuNPs–AgNWs. Here, we have a clearer view of their rough surface, and ∼5 nm in diameter Au nanoparticles were attached to the surface of the AgNWs. The corresponding HRTEM image is shown in Fig. 1F. To further investigate the elemental distribution of AuNPs–AgNWs, EDS elemental mapping was captured. As shown in Fig. 1G and H, the distribution of Au and Ag was homogeneous, which indicated that AuNPs were uniformly adsorbed on the surface of the AgNWs. Thus, there was no doubt that the as-prepared AuNPs–AgNWs were alloyed. The EDS spectrum of the nanowires also demonstrated that the nanowires were composed of Ag and Au besides C and Cu, which were from the remaining reagent and copper net loaded with the nanowires.

To investigate the SERS effect of AuNPs–AgNWs, Raman spectra of 4-MBA and 4-MBA labeled AuNPs–AgNWs were recorded, as shown in Fig. 1J. Two dominant peaks at 1080 and 1587 cm^−1^ were observed for 4-MBA, which come from the aromatic ring vibrations.^28^ 4-MBA labeled AuNPs–AgNWs showed strong SERS signals indicating that the AuNPs–AgNWs had a strong surface enhancement effect. In contrast, 0.1 M 4-MBA could only show a weak SERS signal. Besides, the enhancement factor (EF) of the AuNPs–AgNWs was calculated by using the following expression, EF = (*I*_SERS_/*C*_SERS_)/(*I*_RS_/*C*_RS_), where *I*_SERS_ corresponds to the SERS intensity obtained for the AuNPs–AgNWs colloidal dispersion at a certain concentration *C*_SERS_ of the analyte, and *I*_RS_ corresponds to the Raman intensity obtained under non-SERS conditions at an analyte concentration *C*_RS_. During the experimental process, the AuNPs–AgNWs colloidal dispersion was mixed with the same volume of 4-MBA solution (2 × 10^−6^ M) for 2 h, giving a final concentration of 10^−6^ M. When *C*_SERS_ was 1 × 10^−6^ M and *C*_RS_ was 0.1 M, we got a value of EF = 5.03 × 10^6^, which was much higher than that of gold nanospheres (3 × 10^4^).^29^ The significant enhancement might be due to the nanoparticle–nanowire junctions that potentially act as “hot spots”.

### 3.2 Fabrication of AuNPs–AgNWs substrates

**Fig. 2:**
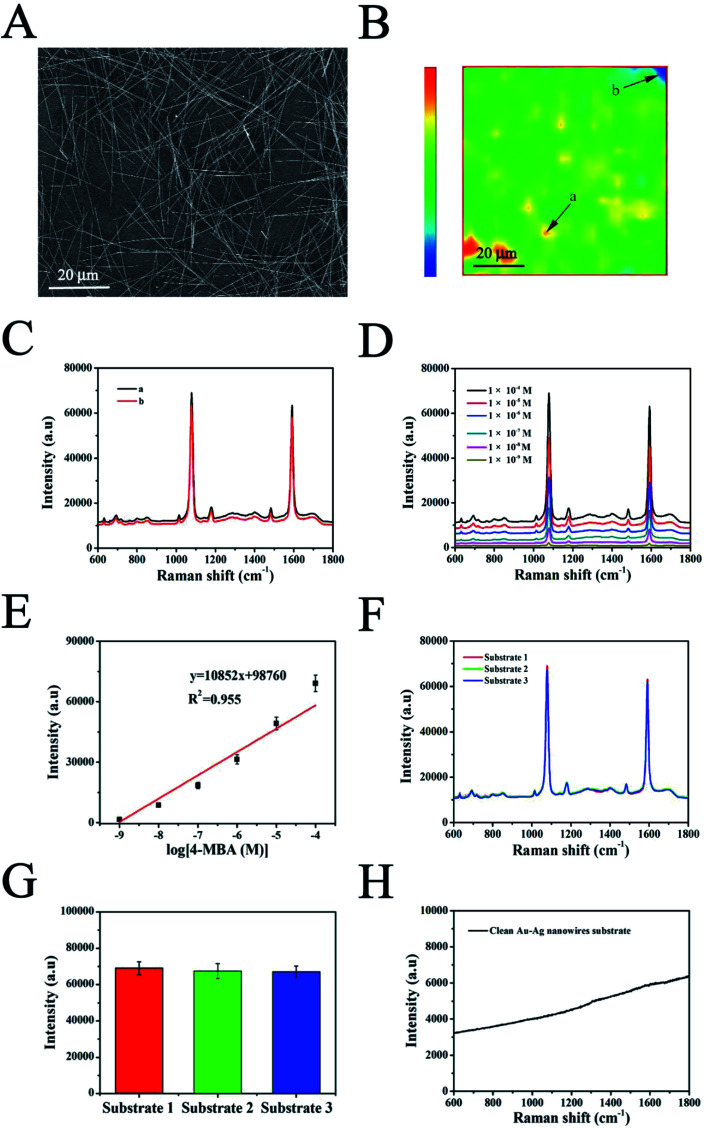
(A) SEM image of the AuNPs–AgNWs substrate. (B) SERS mapping of 4-MBA (1 × 10^−4^ M) at 1080 cm^−1^ using the AuNPs–AgNWs substrate, on which two dots, a and b, were selected. (C) Raman spectra of 4-MBA investigated at the dots mentioned in part B. (D) Raman spectra of the AuNPs–AgNWs substrate measured with different concentrations of 4-MBA (1 × 10^−9^, 1 × 10^−8^, 1 × 10^−7^, 1 × 10^−6^, 1 × 10^−5^, and 1 × 10^−4^ M). (E) The calibration curve of SERS intensities *via* the logarithm values of 4-MBA concentration from 1 × 10^−9^ M to 1 × 10^−4^ M. (F) The reproducibility of the AuNPs–AgNWs substrate measured with 4-MBA (1 × 10^−4^ M). (G) A bar charts of the SERS intensities of the band at 1080 cm^−1^ measured using the substrate mentioned in part F. (H) Raman spectrum of the clean AuNPs–AgNWs substrate.

The AuNPs–AgNWs substrate was fabricated using an electrostatically assisted APTES-functionalized surface-assembly method (Scheme 1), which does not need complicated equipment and is prevalent in commercial manufacture. The aminosilane groups of APTES can boost the adsorption of nanoparticles with an inherent negative charge, so it was employed to functionalize the silicon wafers assisted by electrostatic interaction for adsorption of AuNPs–AgNWs layers. Fig. 2A shows the SEM images of the AuNPs–AgNWs substrate. AuNPs–AgNWs show slight aggregation and are uniformly distributed on the surface of the silicon wafer. To characterize the uniformity of the surface SERS signal, the Raman intensity at 1080 cm^−1^ was mapped over an area of 70 × 70 μm^2^ across the AuNPs–AgNWs substrate after the adsorption of 4-MBA (1 × 10^−5^ M), which is shown in Fig. 2B. The colors of SERS mapping were used to display the intensity of the 1080 cm^−1^ characteristic peak at each grid point according to a color scheme ranging from blue (lowest intensity) to green, yellow, orange, and red (highest intensity). The color of SERS mapping was basically the same, indicating that the SERS enhancement effect of the AuNPs–AgNWs substrates was uniform on the whole. As Fig. 2C shows, there was not much difference in the intensity between the strongest dot (Fig. 2B(a)) and the weakest dot (Fig. 2B(b)), with deviation of less than 8%.

The SERS performance of the fabricated AuNPs–AgNWs substrate was investigated. Fig. 2D shows the SERS spectra of 4-MBA at different concentrations (1 × 10^−9^ M to 1 × 10^−4^ M) while using AuNPs–AgNWs to fabricate the SERS substrate. As shown, different concentrations of the 4-MBA labeled AuNPs–AgNWs substrate all displayed characteristic peaks. When the concentration of 4-MBA was lower than 1 × 10^−9^ M, the AuNPs–AgNWs substrates did not show any SERS signal, thus a concentration of 1 × 10^−9^ M was deemed the limit of detection for the 4-MBA molecules. To examine the correlation of SERS intensity and the concentration, the peak intensities at 1080 cm^−1^ were compared at different concentrations of 4-MBA. As shown in Fig. 2E, the Raman signal increased as the concentration increased, and a logarithmically linear relationship between the SERS intensities and 4-MBA concentration was found. By analyzing the intensities of the band at 1080 cm^−1^, we found that there was a linear relationship between the intensities and the concentration of 4-MBA from 1 × 10^−9^ M to 1 × 10^−4^ M. The linear regression equation was obtained as *y* = 10852*x* + 98 760, with a correlation coefficient of 0.955. The lowest detectable concentration of 4-MBA molecules is 0.93 × 10^−10^ M. Besides uniformity and sensitivity, reproducibility of the substrates is another important property for SERS detection. We made three SERS substrates at different times fabricated with the method mentioned above. The SERS spectra of 4-MBA at one randomly chosen spot on these three substrates are presented in Fig. 2F, and they show no obvious difference. As shown in Fig. 2G, the SERS intensities of the bands at 1080 cm^−1^ corresponding to Fig. 2F were compared. The deviation of the peak intensity at 1080 cm^−1^ was 4.3%, indicating that the substrates fabricated with AuNPs–AgNWs showed good reproducibility. In Fig. 2H, the spectrum of the unlabeled AuNPs–AgNWs substrate showed no obvious characteristic peaks, and this suggested that the substrate had a clean background in the SERS spectrum. Taken together, it can be concluded that the clean AuNPs–AgNWs substrates with ultrahigh sensitivity, homogeneous SERS activity and high reproducibility can be prepared and used as promising candidates for the label-free detection of cellular samples.

### 3.3 Identification of 293T-Mig-2C9 and 293T-Mig-R1 cells

**Fig. 3:**
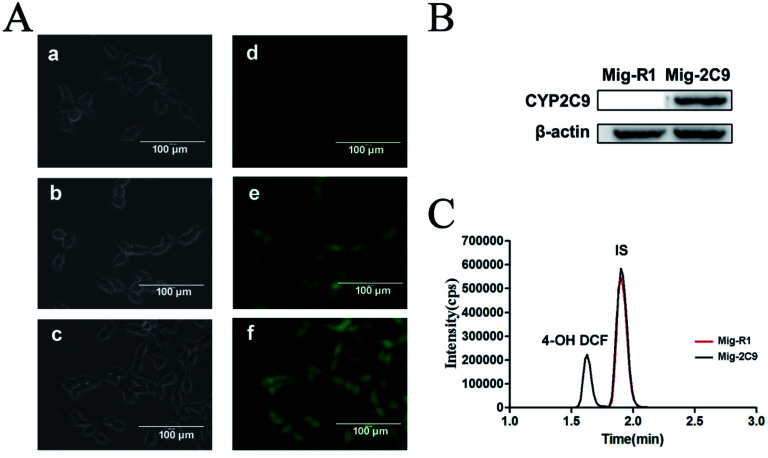
Characterization of CYP2C9 expression. (A) Microscopic morphology and eGFP fluorescence of HEK293T (a and d), 293T-Mig-R1 (b and e) and 293T-Mig-2C9 (c and f) cells. (B) CYP2C9 protein expression was analyzed by western blotting assay. (C) 4′-OH diclofenac was detected with UPLC/MS/MS to evaluate the catalytic activity of CYP2C9.

CYP2C9 constantly expressed HEK293T cells (293T-Mig-2C9 cells) and the empty plasmid control cells (293T-Mig-R1 cells) were constructed by using MigR1 retroviral vector. The morphology and CYP2C9 expression in the two cell lines were analyzed. As shown in Fig. 3A, both 293T-Mig-R1 and 293T-Mig-2C9 showed eGFP fluorescence, while native HEK293T cells exhibited no fluorescence. Morphological changes were not observed in the 293T-Mig-R1 and 293T-Mig-2C9 cells compared with the HEK293T cells. Overexpression of CYP2C9 was observed in the 293T-Mig-2C9 cells (Fig. 3B). Using diclofenac as a probe substrate, CYP2C9 catalytic activity was detected in the 293T-Mig-2C9 cells (Fig. 3C). The results demonstrated that CYP2C9 with catalytic activity was expressed in 293T-Mig-2C9 cells, which provided a model for CYP2C9 function investigation.

### 3.4 *In vitro* cytotoxicity study

**Fig. 4:**
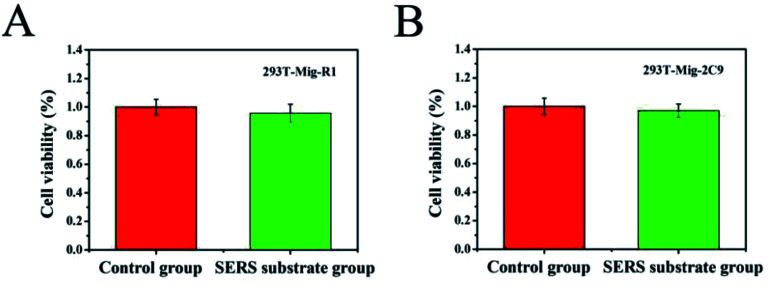
The detection of the cell viability of 293T-Mig-R1 (A) and 293T-Mig-2C9 cells (B) incubated with AuNPs–AgNWs substrates for 24 h.

Assessing the level of toxicity of nanomaterials is of paramount importance in the study of living cells. To examine the feasibility of the AuNPs–AgNWs substrates for application in the detection of cells, their cytotoxicity on 293T-Mig-R1 and 293T-Mig-2C9 cells was explored with a CCK-8 kit. Fig. 4 shows the viability of 293T-Mig-R1 and 293T-Mig-2C9 cells cultured in media with or without Au–Ag nanowire substrates. Compared with the control group, the cell viability of the two cell lines cultured with Au–Ag nanowires substrates decreased slightly. However, the difference was not significant (*P* < 0.05). Thus, the AuNPs–AgNWs substrates have no significant toxicity and are suitable for cellular application.

### 3.5 SERS measurement

**Fig. 5:**
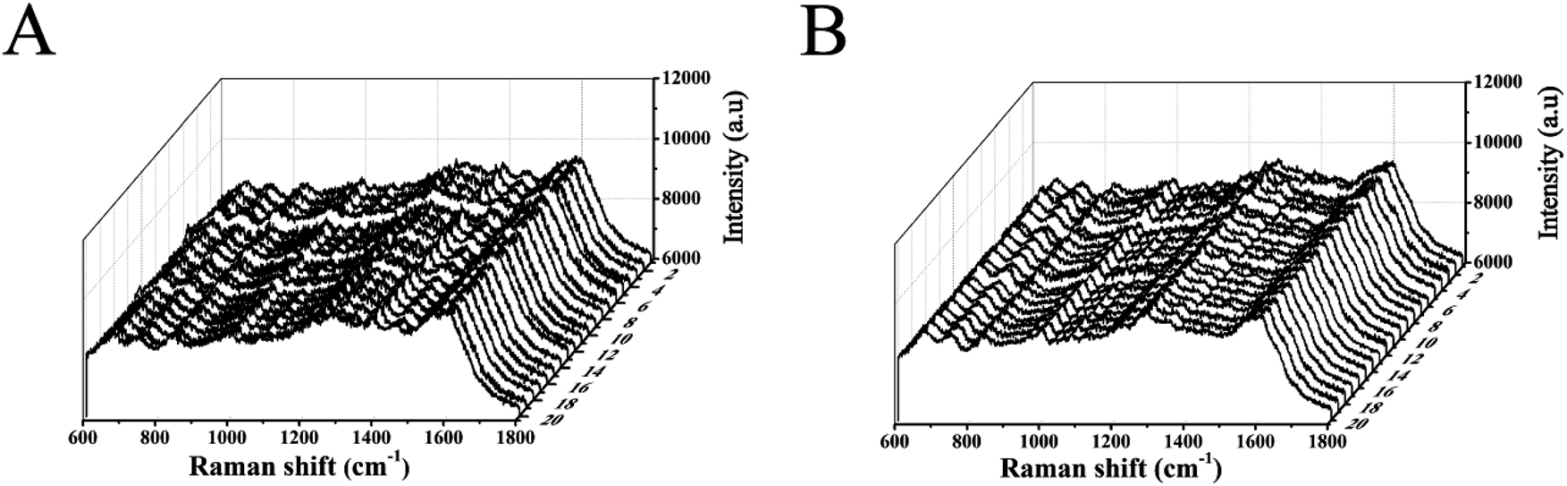
SERS spectra of 20 individual (A) 293T-Mig-R1 and (B) 293T-Mig-2C9 cells incubated with Au–Ag nanowires substrates.

As a direct detection method, AuNPs–AgNWs SERS substrates were incubated with cells to obtain their rich molecular and structural information. After the incubation of cells (293T-Mig-R1 and 293T-Mig-2C9 cells) with AuNPs–AgNWs substrates for 24 h, SERS spectra were collected under experimental conditions (785 nm laser line, 10 s integration times). Fig. 5 shows the SERS spectra of 20 cells of each cell line. The cells incubated with AuNPs–AgNWs substrates for 24 h showed strong Raman signals, and their spectra show many sharp bands ranging from 600 to 1800 cm^−1^. The characteristic peaks of the SERS spectra were capable of providing direct cellular fingerprint information on the structural and chemical composition of these cells. Though lots of researchers have tried to assign peaks to definitive molecules or bonds, progress is still tentative and we hope that our work might help progression in this field. The SERS spectra of different cells in each cell line are almost identical, indicating that these cells possessed very similar cellular components. The results illustrate the ability of SERS to directly detect living cells based on AuNPs–AgNWs substrates.

**Fig. 6:**
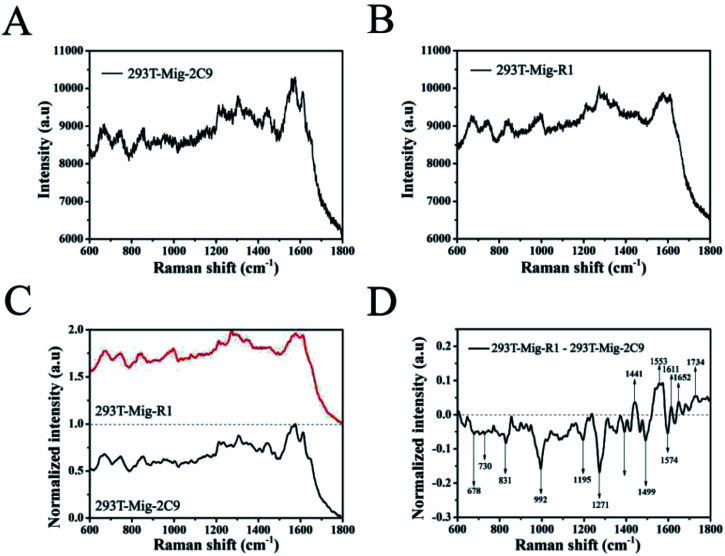
The mean SERS spectra of 293T-Mig-R1 (A) and 293T-Mig-2C9 cells (B) obtained by computing the spectra in Fig. 5. (C) The normalized mean SERS spectra of 293T-Mig-R1 and 293T-Mig-2C9 cells. (D) The difference spectrum of 293T-Mig-R1 and 293T-Mig-2C9 cells.

Then, the SERS spectrum for each group was obtained by averaging over 20 different cells. Fig. 6A and B show the mean SERS spectra of 293T-Mig-R1 and 293T-Mig-2C9 cells. By reference to the Raman band assignments in Table 1,^30–42^ we assigned the characteristic bands of the two cell lines. Important components such as proteins, nucleic acids, lipids, and carbohydrates could be identified in the SERS spectrum of the 293T-Mig-R1 cells. For example, 678 cm^−1^ (ring breathing modes in the DNA bases, G (ring breathing modes in the DNA bases)), 730 cm^−1^ (T (ring breathing mode of DNA/RNA bases)), 853 cm^−1^ (ring breathing mode of tyrosine and C–C stretch of proline ring; glycogen), 904 cm^−1^ (glucose, monosaccharides (β-glucose)), 948 cm^−1^ (single bond stretching vibrations for the amino acids proline and valine and polysaccharides), 1004 cm^−1^ (phenylalanine of collagen, *ν*_s_(C–C), symmetric ring breathing, phenylalanine), 1081 cm^−1^ (carbohydrate residues of collagen), 1155 cm^−1^ (glycogen, C–C, C–N stretching (protein)), 1191 cm^−1^ (nucleic acids and phosphates, aromatic C–O and C–N), 1209 cm^−1^ (tryptophan and phenylalanine *ν*(C–C_6_H_5_) mode), 1271 cm^−1^ (amide III band in proteins, typical phospholipids), 1306 cm^−1^ (CH_3_/CH_2_ twisting or bending mode of lipid/collagen), 1365 cm^−1^ (tryptophan), 1404 cm^−1^ (*ν*(C=O)O^−^ (amino acids aspartic & glutamic acid)), 1442 cm^−1^ (fatty acids, cholesterol and its esters, triglycerides (fatty acids), CH_2_ and CH_3_ bending in lipids), 1553 cm^−1^ (*ν*(C=C), tryptophan (protein assignment), *ν*(C=C), porphyrin), 1574 cm^−1^ (bound and free NADH), 1611 cm^−1^ (cytosine (NH_2_)), 1652 cm^−1^ (lipid (C=C stretch)), and 1734 cm^−1^ (C=O stretching (lipids)). The SERS spectrum of 293T-Mig-2C9 cells displayed the most abundant chemical information from cellular to molecular. Raman bands associated with nucleic acids were located at 678 cm^−1^, 730 cm^−1^, 831 cm^−1^ (asymmetric O–P–O stretching, tyrosine) and 1334 cm^−1^ (CH_3_CH_2_ twisting and wagging in collagen, cellular nucleic acids, CH_3_CH_2_ deforming modes of collagen and nucleic acids). More Raman signatures of proteins were located at 992 cm^−1^ (C–O ribose, C–C), 1081 cm^−1^, 1209 cm^−1^, 1271 cm^−1^, 1611 cm^−1^ and so on. Raman bands assigned to lipids and carbohydrates were located at 904 cm^−1^, 1155 cm^−1^, 1306 cm^−1^, 1442 cm^−1^, and 1574 cm^−1^.

**Table 1:** Raman band assignments^a^

293T-Mig-2C9	293T-Mig-R1	Band (cm^−1^)	Assignment
▲	▲	678	Ring breathing modes in the DNA bases, G (ring breathing modes in the DNA bases)
▲	▲	730	T (ring breathing mode of DNA/RNA bases)
▲		831	Asymmetric O–P–O stretching, tyrosine
	▲	853	Ring breathing mode of tyrosine and C–C stretch of proline ring; glycogen
▲	▲	904	Glucose, monosaccharides (β-glucose)
	▲	948	Single bond stretching vibrations for the amino acids proline and valine and polysaccharides
▲		992	C–O ribose, C–C
	▲	1004	Phenylalanine of collagen, *ν*_s_(C–C), symmetric ring breathing, phenylalanine
▲	▲	1081	Carbohydrate residues of collagen
▲	▲	1155	Glycogen, C–C, C–N stretching (protein)
	▲	1191	Nucleic acids and phosphates, aromatic C–O and C–N
▲	▲	1209	Tryptophan and phenylalanine *ν*(C–C_6_H_5_) mode
▲	▲	1271	Amide III band in proteins, typical phospholipids
▲	▲	1306	CH_3_/CH_2_ twisting or bending mode of lipid/collagen
▲		1334	CH_3_CH_2_ twisting and wagging in collagen, cellular nucleic acids, CH_3_CH_2_ deforming modes of collagen and nucleic acids
	▲	1365	Tryptophan
	▲	1404	*ν*(C=O)O^−^ (amino acids aspartic and glutamic acid)
▲	▲	1442	Fatty acids, cholesterol and its esters, triglycerides (fatty acids), CH_2_ and CH_3_ bending in lipids
	▲	1553	*ν*(C=C), tryptophan (protein assignment), *ν*(C=C), porphyrin
▲	▲	1574	Bound and free NADH
▲	▲	1611	Tyrosine
	▲	1652	Lipid (C=C stretch)
	▲	1734	C=O stretching (lipids)

a Band assignments are based on the literature.^30–42^

To better understand the differences between 293T-Mig-R1 and 293T-Mig-2C9 cells, the mean SERS spectra of the two cell lines were smoothed and normalized, and their difference spectra were compared by subtracting the normalized mean spectrum of 293T-Mig-R1 from the normalized mean spectrum of 293T-Mig-2C9 (Fig. 6C and D). From Fig. 6D, we could learn that the negative bands at 678, 730, 831, 992, 1191, 1271, 1404, 1574, and 1734 cm^−1^ were from the spectrum of 293T-Mig-2C9, indicating that the relative intensity of these bands that were mainly the vibration peaks of DNA, RNA and proteins was higher in the spectrum of 293T-Mig-2C9 when compared with that of 293T-Mig-R1. 293T-Mig-2C9 cells expressed a high level of CYP2C9. The elevation of nucleic acid and protein signals may be caused by the genes and proteins of CYP2C9 in the 293T-Mig-2C9 cells. Whereas, the positive bands in Fig. 6D at 1442 cm^−1^, 1553 cm^−1^, 1611 cm^−1^, and 1652 cm^−1^ were speculated to be from the spectrum of 293T-Mig-R1. The 1553 cm^−1^ band was related with the signal of porphyrin. CYP2C9 has a heme (a porphyrin complex) containing catalytic center. We suspected that the incorporation of heme into the CYP2C9 catalytic center may account for the decrease of the 1553 cm^−1^ signal in the 293T-Mig-2C9 cells.^43^ The Raman band at 1442 cm^−1^ represents fatty acids, cholesterol and its esters, triglycerides (fatty acids) and CH_2_ and CH_3_ bending in lipids. It was reported that arachidonic acid (AA), a fatty acid that is usually esterified in membrane phospholipids in cells, was a substrate of CYP2C9.^44^ The substrates of CYP2C9 also include steroid hormones that are synthesized from cholesterol.^45^ The catalytic activity of CYP2C9 may be responsible for the alteration at 1442 cm^−1^. In addition, CYP2C9 will attack double bonds in AA, and catalyze them into epoxide forms, which may account for the change of the 1652 cm^−1^ signal. However, further investigations are still required for better understanding the shift of the Raman spectrum.

### 3.6 PCA

**Fig. 7:**
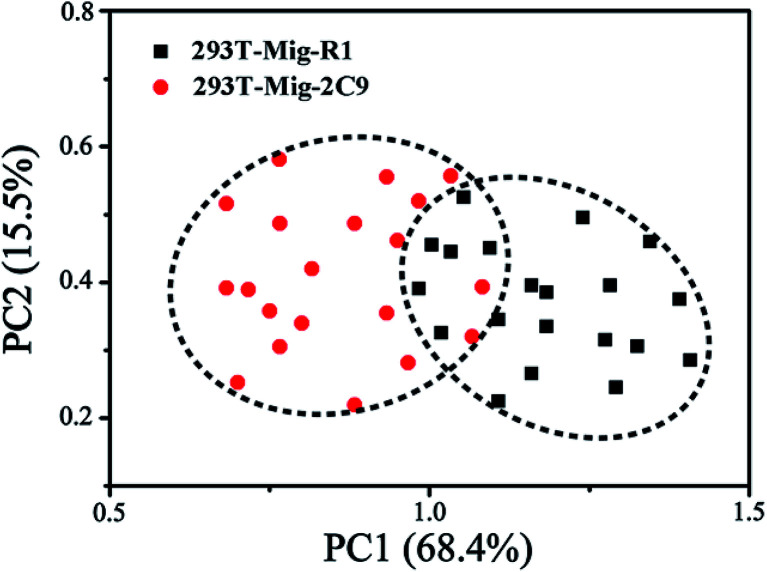
PCA score plots of SERS spectra for 293T-Mig-R1 and 293T-Mig-2C9 cells. Each spot represents one cell and each cell type is coded by different colors and shapes.

PCA, a powerful multivariate analytical tool, was employed to provide the quantitative determination of cells amongst non-negligible spectroscopic variations. PCA is a statistical method for finding the components in a multivariate data set that have the largest variance. It serves to reduce the dimension of the data to a few key components by orthogonal transformation for the purpose of accentuating the differences and commonalities across data sets.^46^ It is capable of identifying characteristics that relate to classification and discrimination of two groups of data. In order to determine if the SERS spectra could be used to distinguish between the two types of cells, we employed PCA for analysis of their spectral data. As shown in Fig. 7, using principal component scores PC1 (68.4% variation) and PC2 (15.5% variation), the scatter plots of SERS spectra for each cell line of about twenty cells were projected into the two dimensional images. Apparently, the PCA plots indicated that the SERS spectra of 293T-Mig-R1 cells and 293T-Mig-2C9 cells were distinguishable into two distinct groups. The plotted group of 293T-Mig-R1 cells only has a small overlap with that of the 293T-Mig-2C9 cells. This result was consistent with the SERS analysis mentioned above. This could be deduced from the spectral differences between the two kinds of cells as spectral differences represent the differences in the composition of proteins, DNA, RNA, carbohydrates and lipids. 293T-Mig-2C9 cells have more nucleic acids and proteins but have fewer lipids and some amino acids than 293T-Mig-R1 cells. However, there are also some similar biochemical compositions between the two kinds of cells. PCA gives more intuitive results than direct analysis of the spectral difference between 293T-Mig-R1 and 293T-Mig-2C9 cells. The results suggested that PCA was able to achieve a successful segregation of CYP2C9-expressing from non-expressing cells based on their specific spectra.

## 4. Conclusions

This work demonstrated the fabrication of a highly sensitive and uniform AuNPs–AgNWs SERS substrate for the label-free detection of and discrimination between 293T-Mig-R1 and 293T-Mig-2C9 cells. The successful synthesis of closely packed and long AuNPs–AgNWs in high yields was achieved by a facile electrostatic interaction method. Then, AuNPs–AgNWs were utilized to assemble the SERS substrates, and these substrates were measured with different concentrations of 4-MBA molecules. Their limit of detection was 1 × 10^−9^ M. The intensity of the peak at 1080 cm^−1^ demonstrated the good reproducibility of the fabricated SERS substrates, producing RSDs of less than 10% in the different substrates. Additionally, CCK8 assays indicated the low cytotoxicity of the SERS substrates. The AuNPs–AgNWs substrates enabled us to successfully detect the SERS spectra of 293T-Mig-R1 and 293T-Mig-2C9 cells. The difference spectra showed that the 293T-Mig-R1 cells had more abundant lipids and some amino acids compared to 293T-Mig-2C9 cells, while their nucleic acid and some protein component content values were lower than the 293T-Mig-2C9 cells. However, there was no significant difference in carbohydrates between the two cell lines. PCA further demonstrated that Au–Ag nanowire substrates could achieve successful segregation of 293T-Mig-R1 and 293T-Mig-2C9 cells, which paves the way to applications in the diagnosis of diseases in the future.

## Conflicts of interest

There are no conflicts to declare.

## Supplementary Material
